# Treatment of Rheumatoid Arthritis with Marine and Botanical Oils: An 18-Month, Randomized, and Double-Blind Trial

**DOI:** 10.1155/2014/857456

**Published:** 2014-03-19

**Authors:** George W. Reed, Katherine Leung, Ronald G. Rossetti, Susan VanBuskirk, John T. Sharp, Robert B. Zurier

**Affiliations:** ^1^University of Massachusetts Medical School, Department of Medicine, Division of Preventive and Behavioral Medicine, 55 Lake Avenue North, Shaw Building, Worcester, MA 01655, USA; ^2^University of Massachusetts Medical School, Department of Medicine, Rheumatology Division, 55 Lake Avenue North, LRB 240, Worcester, MA 01655, USA; ^3^University of Massachusetts Medical School, Department of Family Medicine and Community Health, 55 Lake Avenue North, Benedict Building A3-214, Worcester, MA 01655, USA; ^4^University of Washington School of Medicine, Bainbridge Island, Seattle, WA, USA; ^5^University of Massachusetts Medical School, Department of Medicine, Rheumatology Division, 55 Lake Avenue North, LRB 840D, Worcester, MA 01655, USA

## Abstract

*Objective*. To determine whether a combination of borage seed oil rich in gamma linolenic acid (GLA) and fish oil rich in eicosapentaenoic acid (EPA) and docosahexaenoic acid (DHA) is superior to either oil alone for treatment of rheumatoid arthritis (RA). * Methods*. Patients were randomized into a double-blind, 18-month trial. Mixed effects models compared trends over time in disease activity measures. * Results*. No significant differences were observed in changes in disease activity among the three randomized groups. Each group exhibited significant reductions in disease activity (DAS28) at 9 months (fish: −1.56[−2.16, −0.96], borage: −1.33[−1.83, −0.84], combined: −1.18[−1.83, −0.54]) and in CDAI (fish: −16.95[−19.91, −13.98], borage: −11.20[−14.21, −8.19], and combined: −10.31[−13.61, −7.01]). There were no significant differences in change of RA medications among the three groups. Reduced disease activity in study patients was similar to matched patients from an RA registry, and reduction in DMARD use was greater (*P* < 0.03) in study patients. * Conclusion*. All 3 treatment groups exhibited similar meaningful clinical responses after 9 months, improvements which persisted for 18 months, and a response similar to matched patients from an RA registry. Study patients were able to reduce DMARD therapy given in combination with TNF antagonists to a greater extent than registry patients.

*This paper is dedicated to the memory of Dr. John T. Sharp, M.D., a pioneer and innovator in the field of musculoskeletal radiology*

## 1. Introduction

Abundant experimental evidence supports the view that eicosanoids participate in development and regulation of immunological and inflammatory responses [1–4]. Because essential fatty acids are precursors to eicosanoids and are important determinants of cell function, they influence immune responses [5]. A disease such as rheumatoid arthritis (RA), characterized by abnormal immune responses, inflammation, and joint tissue injury [6], may therefore be amenable to control by treatment with oils rich in particular polyunsaturated fatty acids.

Gamma linolenic acid (GLA:  18 : 3 omega 6) is an essential fatty acid found in borage seed oil. GLA is metabolized to dihomogamma linolenic acid (DGLA; 20 : 3 omega 6), the immediate precursor of prostaglandin E_1_ (PGE_1_), an eicosanoid with anti-inflammatory and immunoregulatory properties [7]. In addition, GLA cannot be converted to inflammatory leukotrienes by 5-lipoxygenase. Instead, it is converted to 15-hydroxy DGLA, which has the virtue of suppressing 5-lipoxygenase activity [8]. GLA and DGLA also modulate immune responses in an eicosanoid independent manner by acting directly on T lymphocytes [9], and GLA suppresses acute and chronic inflammation, including arthritis, in animal models [10]. More importantly, in several randomized, placebo-controlled trials in RA patients, GLA in borage or primrose seed oils reduced synovitis and the need for nonsteroidal anti-inflammatory agents [11–13].

Fish oil, rich in eicosapentaenoic acid (EPA; 20 : 5 omega 3) and docosahexaenoic acid (DHA; 22 : 6 omega-3), suppresses formation of the inflammatory eicosanoids PGE_2_, thromboxane A_2_ (TXA_2_), and leukotriene B_4_ (LTB_4_). The LTB_5_ which is produced is a far less potent mediator than LTB_4_. Each of 12 randomized, placebo-controlled, and double-blind trials of fish oil in RA documents clinical improvement, including reductions in duration of morning stiffness, number of tender joints, joint pain, time to fatigue, and increased grip strength. Those studies that monitored NSAID use suggest that fish oil treatment has an NSAID sparing effect [14, 15].

A combination of EPA and GLA enriched oils exhibits synergy in reduction of synovitis in animal models [16], and treatment of RA patients with black currant seed oil, which contains both the n-3 fatty acid alpha linolenic acid (converts to EPA) and the n-6 GLA, suppresses synovitis in these patients [17]. These results suggest that a combination of GLA and EPA may be a more useful therapy for RA than each fatty acid alone. Therefore, we carried out a phase 3 trial of borage seed oil, fish oil, and a combination of the two oils in patients with RA and active synovitis, to determine whether the combination is superior to treatment with either oil alone.

## 2. Study Design

The study was an 18-month randomized, double-blind comparison of borage seed oil, fish oil, and the combination of both oils in RA patients with synovitis. Patients were evaluated at 3-month intervals. The protocol was reviewed and approved by the Committee for the Protection of Human Subjects in Research at the University of Massachusetts Medical School and by the Food and Drug Administration. Subsequent approvals were obtained from Review Boards at the University of Alabama, Geisinger Clinic, Fallon Health Care, and the New England IRB. Written informed consent was obtained from each patient.

## 3. Eligibility

Patients were eligible to participate in the study if they had RA according to the 1987 criteria of the American Rheumatism Association [18], were in functional class I, II, or III according to the revised criteria of the American College of Rheumatology [19], and were between the ages of 18 and 85. Patients had active disease as manifest by at least 3 swollen joints and 6 tender joints at the time of enrollment. In addition, patients had an erythrocyte sedimentation rate (ESR) of >28 mm/hr or morning stiffness of at least 45 min. Patients were on a stable dose of disease modifying antirheumatic drugs (DMARDs) and/or biologic agents for at least 2 months before the screening visit, with a total duration of therapy of at least 6 months. An NSAID dose (with or without other treatment) was stable for at least one month before screening, and a prednisone (or equivalent corticosteroid) dose ≤10 mg/d was stable for at least one month before screening.

Patients were ineligible for the study if they had been treated with investigational drugs within one month of entry. If a patient was taking a fish oil or borage oil supplement, the dose was stable and ≤2000 mg/d for each supplement for 2 months before screening. An intra-articular corticosteroid injection within 6 months of screening excluded that joint from evaluation until 6 months after injection. An aspartate transaminase (AST) or alanine transaminase (ALT), or creatinine > 1.5 times upper limit of normal, or a total bilirubin > 1.8 mg/dL excluded patients.

## 4. Treatment

Patients were randomized to receive either 6 borage seed oil (1.8 gm GLA) capsules plus 7 sunflower seed oil capsules daily, or 7 fish oil (2.1 gm EPA/1.4 gm DHA) capsules and 6 sunflower seed oil capsules daily, or 6 borage seed oil capsules plus 7 fish oil capsules daily. All capsules were identical in appearance and color and were purchased from Bioriginal Food and Service Corp., Saskatoon, Canada, who shipped the capsules in coded opaque plastic bottles to the University of Massachusetts University Hospital Pharmacy, from whence they were distributed to participating centers. Capsules were taken in 2 or 3 divided doses with meals.

## 5. Clinical Assessment

Each individual patient was evaluated by the same examiner at each visit. The 4-value modified disease activity score using ESR (DAS 28) was the primary outcome measure.

The DAS 28 has been validated [20] and is as representative of change in disease status as the ACR criteria [21]. A validated [22] Clinical Disease Activity Index (CDAI), omitting acute phase reactants, was also used. The CDAI is valid for assessment of RA activity and treatment response [23]. Patients were evaluated at baseline and 3-month intervals.

Patients were instructed to maintain their typical diet. Dietary 24 hr recalls were collected at baseline and 18 months, or the terminal visit.

## 6. Evaluation of Radiographs

Radiographs of hands and feet were obtained at baseline and 18 months, or early exit, unless the patient exited before 12 months. All available radiographs were scored for erosions (scale 0–5) and joint space narrowing (0–4) by Dr. John T Sharp. Erosion and narrowing scores were summed to establish a total score. Radiographs were evaluated in patient sets without knowledge of the order taken or treatment received by the patient. Films were scored as described [24]. A total of 17 joints in each hand and wrist and 6 joints in each forefoot were scored for erosions and narrowing. Maximum possible scores were 230 for erosions and 184 for narrowing. Patients were categorized as to whether they exhibited radiographic evidence of progressive or nonprogressive disease [25].

## 7. Data Management and Statistical Analysis

**Figure 1:**
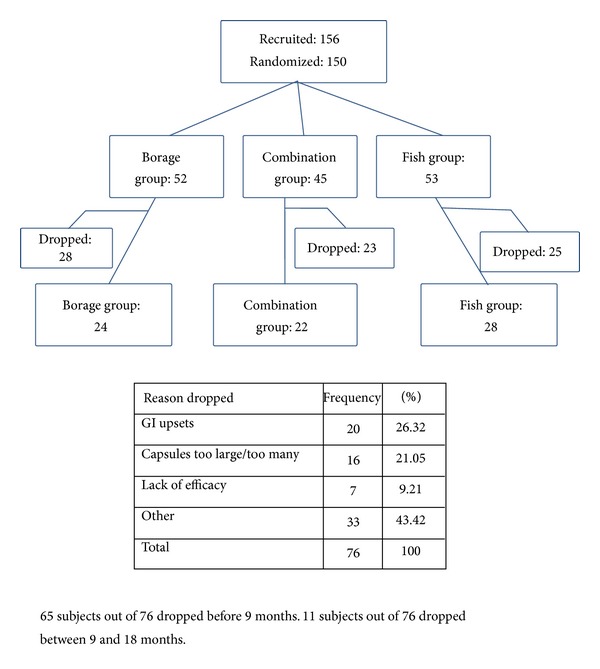
Flow chart: patient recruitment and progress.

A sample of 45 patients per group was needed for 80% power for a difference of 0.6 in change in DAS between groups. There were 156 patients screened as eligible and 150 patients were randomized. At 9 months there were approximately 31 per group and at 18 months 26 per group. The number of patients completing 18 months is shown in Figure 1. First screening visit was in 11/2004 and last follow-up visit was in 5/2008.

### 7.1. Statistical Methods

Baseline characteristics were compared among groups using analysis of variance (ANOVA) for continuous covariates and Chi-square tests for dichotomous covariates. For skewed continuous distributions a Kruskal-Wallis test was used and for small cell sizes Fisher's exact test. Changes in DAS and CDAI were tested over time using linear random effects models with patient random intercepts and slope. DAS and CDAI were modeled as a function of randomized group and time, and an interaction term of group and time was used to test if trends were different by group. For DAS, time was used as a categorical variable (3 time points). For CDAI (7 time points), time was continuous and estimated lowess curves [26] indicated a quadratic association. Mean changes from baseline in DAS and CDAI were based on the models.

Sensitivity analyses were done, using completers (to 18 months) and two imputations: (1) dropped patients return to baseline values and (2) multiple imputation for missing outcomes [27–29]. Results between groups from all analyses were similar. Statistical comparisons of trends among groups were consistent in all models.

Use of TNF antagonists, NSAIDS, and corticosteroids was analyzed using logistic regression.

Change in X-ray scores between baseline and follow-up was compared among groups using a Kruskal-Wallis test. Changes in scores were compared to changes in DAS and CDAI using Spearman correlations. Analyses examined the absolute change and rate of change, and results from each analysis were similar. Rate of change estimates are presented.

Patients in this study were matched to patients in the Consortium of Rheumatology Researchers of North America (CORRONA), a large RA registry [30], selected to meet the criteria for this study. We matched patients on the components of the CDAI so disease activity was similar at baseline. Trends in CDAI over time were compared between the matched groups (patients in this study and CORRONA patients) using random effects models.

## 8. Results

Among eligible patients, 150 were randomized. Patients randomized to each group and the numbers completing the 18-month study are listed in Figure 1 including reasons for dropouts. Given that beneficial effects of the oils can occur 2-3 months after onset of therapy, outcomes were evaluated for patients in the trial for at least 12 weeks (138/150 = 92%). The overall dropout rate (<18 months in the study) was 51% and was not significantly different among the groups (borage: 54%; fish: 47%; combination 51%, *P* = 0.79). The largest proportion dropped due to size and number of capsules or because of gastrointestinal problems (47%).

Randomized groups were balanced across all characteristics (*P* ≥ 0.2 for all characteristics) (Table 1).

Baseline characteristics between completers (18 months) and those who dropped were compared. The completers were more likely to be male (24% versus 13%) and African-American (11% versus 1%) and have lower patient global scores (4.31 versus 5.38) and physician global scores (4.24 versus 4.98) but higher swollen joint counts (14.12 versus 12.17) at the beginning of the study. CDAI and DAS were not different (*P* > 0.76).

Compliance, assessed by capsule counts and patient report, indicates that 45% of patients reported ever missing a dose (borage: 42%, fish 48%, and combination 47%, *P* = 0.77). Median total capsules missed (excluding those with 0) were 182 (borage: 164, fish 169, and combination 256, *P* = 0.65). Comparison of change in CDAI and DAS adjusted for number of capsules missed did not change the group comparisons.

## 9. Safety

No significant changes in dietary intake of fatty acids or weight change were observed. Total reported adverse events (AE) were comparable with rates of 1.65/person-year in fish, 1.48/person-year in borage, and 1.73/person-year in combined. With fish as the reference group the incident rate ratios (IRR) were borage IRR = 0.9[0.66, 1.23] and combined IRR = 1.05[0.77, 1.43].

If AEs are restricted to events that are “possibly related to treatment,” then event rates are 0.30/person year for fish, 0.46/person year for borage, and 0.77/person year for combined. With fish as the reference group, borage IRR = 1.56[0.82, 2.95] and combined IRR = 2.61[1.44, 4.74]. All related AEs were associated with gastrointestinal distress (all but two were mild to moderate). Two patients dropped due to Grade IV AEs: “burning in the throat” and severe nausea. Both were in the borage group.

## 10. Clinical Responses

No significant differences were observed between groups in trends in DAS over time (*P* = 0.45) (Table 2(a)). Estimated changes in DAS in each group are shown in Table 2(a). When all 3 groups were combined, a significant (*P* < 0.001) reduction of 1.51 in DAS by 18 mo was observed with nearly all the decrease occurring by 9 mo.

The same trends were noted in CDAI. There was no significant difference in trend in CDAI over time among the three groups (*P* = 0.15). Estimated changes in CDAI are shown in Table 2(b). The combined groups exhibited a significant reduction in CDAI (*P* < 0.001) over time and an estimated reduction in CDAI of 12.5 by 9 months.

The distribution of therapy for RA is shown in Table 3. There were no significant differences in therapy by group at 9 months and 18 months. There was no significant difference between groups in change in medications over time.

Based on X-rays at baseline and follow-up (Table 4) there were no significant differences between the 3 groups in the combined scores (*P* = 0.38), erosion scores (*P* = 0.28), or joint space narrowing scores (*P* = 0.27). There was a significant increase in all scores over time. Radiographs from 8 patients (11%) exhibited a reduction in the combined Sharp score, whereas 33 (45%) exhibited an increase, and 32 (44%) showed no change.

Significant associations were not observed between change in X-ray scores and change in DAS (Spearman *r* = 0.20, *P* = 0.13) or CDAI (*r* = 0.06, *P* = 0.63), or with baseline levels of DAS (*r* = −0.01, *P* = 0.98) or CDAI (*r* = −0.02, *P* = 0.86).

**Figure 2:**
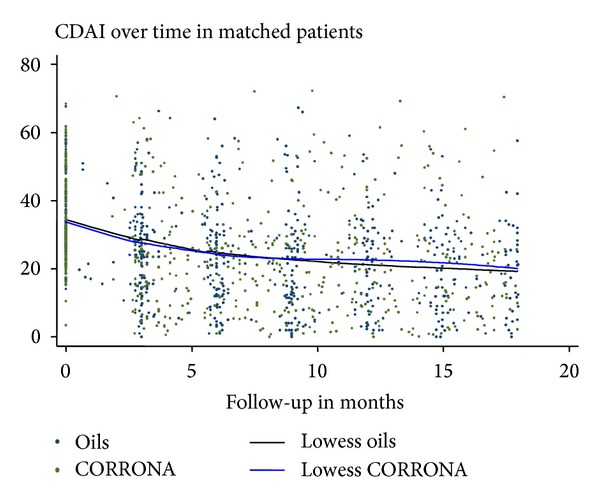
Comparison of OILS Study and matched CORRONA patients. Lowess curve fits of CDAI over time (months) in the study patients (OILS) and a matched set of patients from a rheumatoid arthritis registry (CORRONA).

Trends in CDAI over time in patients in this study were compared to matched patients from the CORRONA registry (Figure 2). No significant difference was seen in trends over time between the two groups (*P* = 0.31).

Medication use was also compared. There were no significant differences in change in prednisone use (*P* = 0.81) or in change in TNF antagonists (*P* = 0.99). There was a significant difference in change in oral DMARD use (*P* = 0.026) between the two groups: patients in this study exhibited a decrease in DMARD treatment (OR = 0.86 for one year, *P* = 0.11), whereas CORRONA patients had an increase in DMARD treatment (OR = 1.20 for one year, *P* = 0.11). This difference manifests itself in the change in combination therapy (TNF antagonist + oral DMARD). Patients treated with the oils reduced their combination therapy (OR = 0.85, *P* = 0.03), but CORRONA patients on TNF antagonists did not reduce DMARD treatment (OR = 1.00, *P* = 0.97).

## 11. Discussion

The premise that the combination of borage and fish oils would be superior to either oil alone is not confirmed by this study. All 3 treatment groups exhibited significant reductions in both DAS (1.5) and CDAI (12.5) at 9 months, improvements which persisted at 18 months. Given the efficacy of both oils as treatment for RA [31] a placebo group was not included. However, comparison with the clinical course in a matched set of patients in the CORRONA Registry receiving similar traditional treatment showed similar disease activity trends. Evidence of a significant reduction in combination therapy (oral DMARD + TNF antagonist) by study patients versus patients from the RA registry suggests that treatment with the oils allowed reduction of treatment with more toxic agents. In randomized, placebo-controlled clinical trials in patients with RA, EPA/DHA and GLA rich oils may replace NSAIDs and do not cause adverse events associated with NSAID administration [32]. Our observations [33] that DGLA suppresses synovial cell proliferation and results of a controlled trial [32] which indicate that RA patients are more improved after 12 months of GLA treatment than after 6 months suggest that GLA might function as a DMARD.

No differences in X-ray progression were observed among the 3 groups. Changes in radiographic scores did not correlate with clinical responses (DAS and CDAI). These findings are in agreement with known clinical observations that some patients who experience substantial improvement in joint pain and swelling exhibit worsening of joints as assessed by radiography and to similar findings from controlled clinical trials [34]. It is likely that agents that reduce inflammatory responses, resulting in reduction of joint pain and swelling, do not influence pathways leading to joint tissue injury.

All treatments were safe. Rates and types of adverse events were similar across all 3 treatment groups and were related almost entirely to the large size and number of capsules and to gastrointestinal distress. Although we have not observed increases in circulating arachidonic acid (AA) after administration of borage seed oil, the possibility must be considered in long term treatment. When fish oil is administered with borage oil to healthy individuals, bioconversion of GLA to AA is prevented [35]. Thus, consideration can be given to the use of one or another or both of these oils for treatment of RA. Although NSAIDs act rapidly, adverse events associated with NSAID use are well known [36]. The delay in symptomatic relief with marine and botanical oils (8–12 weeks) should be acceptable for treatment of a chronic condition such as RA. Neither borage oil nor fish oil is associated with serious gastrointestinal events (ulceration, bleeding, and perforation). In addition, whereas NSAIDs increase the incidence of myocardial infarction and stroke [37], fish oil reduces cardiovascular events in patients at risk, including those with RA [38].

Unfortunately, as is the case for studies in which fish oil was used to reduce cardiovascular events [38], the dropout rate in our trial was in excess of 45%. Much of that problem is due to the large size and number of capsules administered each day. It would be preferable to use isolated fatty acids which can be formulated in higher concentrations in small capsules. Among those patients who did not complete the 18-month trial, a sufficient number remained for at least 12 weeks, to allow evaluation (Table 2).

Patients who completed the entire 18-month trial were mainly male and African American, despite the fact that this group had higher swollen joint counts at baseline. That may reflect the problem of access to health care for people of color in the United States [39]. Patients who struggle to purchase health insurance and traditional medicine may be more likely to stay the course in a study that provides treatment and care.

Although the combination of oils did not prove superior to either oil alone, patients in each group did improve significantly, so that most patients had incentive to continue the trial. In addition, these oils can substitute for NSAIDs, and the combination of both oils allows reduction of more toxic DMARDs by patients treated with agents that block TNFa. Newer formulations which provide appropriate fatty acids in smaller capsules would encourage more patients to use them rather than NSAIDs. Further studies of the combination of marine and botanical oils might provide data to persuade physicians to use them in treatment of patients on DMARDs and biologic agents, in an effort to reduce treatment with the more toxic DMARDs.

## Figures and Tables

**Table 1:** Baseline characteristics in the three randomized groups.

Mean (SD) or median [IQR] (*n* = total) or percent (*c* = num) (*n* = total)	Borage group (*n* = 52)	Combination group (*n* = 45)	Fish group (*n* = 53)	*P* value
Body mass index (kg/m^2^)	29.4 (7.1) (*n* = 49)	30.4 (8.2) (*n* = 45)	31.4 (9.5) (*n* = 51)	0.499
Female	76.9 (*c* = 40)	80.0 (*c* = 36)	86.8 (*c* = 46)	0.415
Age	60.3 (9.2)	60.5 (13.0)	57.3 (12.3)	0.295
Black/African-American	7.8 (*c* = 4) (*n* = 51)	2.2 (*c* = 1) (*n* = 45)	7.8 (*c* = 4) (*n* = 51)	0.428**
Does subject exercise? (yes)	57.7 (*c* = 30)	51.1 (*c* = 23)	50.9 (*c* = 27)	0.738
Rheumatoid factor positive	73.9 (*c* = 34) (*n* = 46)	83.9 (*c* = 26) (*n* = 31)	72.7 (*c* = 32) (*n* = 44)	0.491
Log ESR value	2.9 (1.1) (*n* = 52)	2.7 (1.0) (*n* = 43)	3.05 (1.0) (*n* = 51)	0.199
CRP value	0.6 [0.3–1.5] (*n* = 48)	0.6 [0.4–2.7] (*n* = 40)	0.8 [0.4–1.7] (*n* = 46)	0.415*
Disability index (mHAQ)	0.5 [0.1–0.7] (*n* = 51)	0.5 [0.1–0.9] (*n* = 45)	0.5 [0.1–0.9] (*n* = 53)	0.788*
Clinical Disease Activity Index	34.4 (11.5)	36.0 (12.6)	35.8 (11.3)	0.763
Simplified Disease Activity Index	33.1 (27.0–47.1) (*n* = 48)	34.1 (28.8–50.2) (*n* = 40)	35.5 (30.8–46.2) (*n* = 46)	0.442
Disease activity score	5.0 [4.1–5.8] (*n* = 52)	4.8 [3.9–5.7] (*n* = 43)	5.2 [4.4–6.0] (*n* = 51)	0.404*
DAS remission (<2.6)	3.8 (*c* = 2) (*n* = 52)	4.6 (*c* = 2) (*n* = 43)	2.0 (*c* = 1) (*n* = 51)	0.859**
Total number of tender joints	12.5 [7.5–18.0]	11.0 [7.0–18.0]	12.0 [8.0–20.0]	0.936*
Total number of swollen joints	12.0 [8.0–15.0]	12.0 [8.0–19.0]	12.0 [8.0–18.0]	0.733*
Physician global	3.8 [3.0–5.8]	4.0 [3.0–6.3]	4.1 [3.0–7.0]	0.646*
Patient global	5.0 [3.0–6.7]	5.5 [4.0–7.0]	5.0 [2.8–6.5]	0.435*
Patients taking methotrexate	61.5 (*c* = 32)	68.9 (*c* = 31)	64.1 (*c* = 34)	0.748
Patients taking NSAIDS	28.8 (*c* = 15)	31.1 (*c* = 14)	26.4 (*c* = 14)	0.876
Patients taking corticosteroid	21.1 (*c* = 11)	24.4 (*c* = 11)	28.3 (*c* = 15)	0.696
Patients taking DMARDS	71.1 (*c* = 37)	77.8 (*c* = 35)	69.8 (*c* = 37)	0.647
Patients taking TNF blockers	50.0 (*c* = 26)	44.4 (*c* = 20)	50.9 (*c* = 27)	0.791
Morning stiffness (minutes)	60.0 [30.0–180.0] (*n* = 51)	60.0 [35.0–120.0] (*n* = 45)	60.0 [42.5–120.0] (*n* = 52)	0.439*
Duration of rheumatoid arthritis (years)	7.4 [3.0–15.3] (*n* = 52)	12.2 [4.4–18.0] (*n* = 44)	6.3 [4.2–18.9] (*n* = 51)	0.349*

*P value is from a Kruskal-Wallis test, median with the 25th and the 75th percentile is shown.

**P value is from a Fisher's exact test. All other *P* values come from a *t*-test or chi-square.

**Table:** (a)

	9 months	18 months
Borage group	−1.33 (−1.83 to −0.84)	−1.53 (−2.05 to −1.01)
Combination group	−1.18 (−1.83 to −0.54)	−1.28 (−1.88 to −0.67)
Fish group	−1.56 (−2.16 to −0.96)	−1.45 (−2.01 to −0.89)

**Table:** (b)

Time point	Borage group			Combination group			Fish group		
	Coef.	95% Conf. interval		Coef.	95% Conf. interval		Coef.	95% Conf. interval	
3 months	−6.75	−8.94	−4.57	−6.59	−8.98	−4.21	−10.69	−12.84	−8.54
6 months	−9.26	−12.00	−6.52	−8.81	−11.81	−5.81	−14.37	−17.07	−11.67
9 months	−11.20	−14.21	−8.19	−10.31	−13.61	−7.01	−16.95	−19.91	−13.98
12 months	−12.55	−15.66	−9.45	−11.10	−14.50	−7.69	−18.42	−21.46	−15.37
15 months	−13.34	−16.59	−10.09	−11.16	−14.70	−7.62	−18.78	−21.92	−15.63
18 months	−13.54	−17.31	−9.77	−10.51	−14.57	−6.45	−18.03	−21.60	−14.45

**Table 3:** Distribution of medications at baseline, 9, and 18 months.

	Baseline		9 months		*P* value	18 months		*P* value
	*N*	%	*N*	%		*N*	%	
Using TNF antagonists					0.37			0.36
Borage group	26	50.0	18	58.1		12	48.0%	
Combination group	20	44.4	11	40.7		10	45.4%	
Fish group	27	50.9	16	44.4		19	63.3%	
Using DMARDS					0.46			0.14
Borage group	37	71.1	23	74.2		16	64.0%	
Combination group	35	77.8	21	77.8		19	86.4	
Fish group	37	69.8	26	72.2		19	63.3	
Using corticosteroids					0.47			0.07
Borage group	11	21.1	6	19.3		2	8.0	
Combination group	11	24.4	9	33.3		8	36.4	
Fish group	15	28.3	8	22.2		7	23.3	
Using NSAIDS					0.49			0.19
Borage group	15	28.8	6	19.3		4	16.0	
Combination group	14	31.1	9	33.3		8	36.4	
Fish group	14	26.4	10	27.8		11	36.7	

A comparison of change over time among groups for TNF blockers, DMARD, corticosteroid, and NSAID use showed no significant differences between the groups. TNF blocker *P* = 0.09, DMARDS *P* = 0.38, corticosteroids *P* = 0.32, and NSAIDS *P* = 0.94.

**Table 4:** Radiograph Scores.

Mean (SD) Median [interquartile range]	Baseline	Follow-up	Score change/year	*P* value
Combined score				
Borage *n* = 25	17.8 (29.4) 7.0 [1.0, 19.0]	18.8 (28.8) 7.0 [3.0, 27.0]	0.69 (2.3)	*P* = 0.38*
Combination *n* = 18	49.2 (54.6) 27 [10.0, 94.0]	50.8 (56.8) 26.5 [10.0, 95.0]	1.07 (3.0)	
Fish *n* = 30	23.1 (33.1) 7.0 [0.0, 33.0]	26.5 (35.5) 8.0 [0.0, 41.0]	2.27 (6.3)	
All groups	27.7 (39.9) 9.0 [1.0, 39.0]	29.8 (41.3) 11.0 [3.0, 41.0]	1.44 (4.5)	*P* < 0.001^+^
Erosions score				
Borage	6.5 (11.4) 2.0 [0.0, 8.0]	7.1 (11.3) 4.0 [1.0, 8.0]	0.44 (1.3)	*P* = 0.28*
Combination	22.9 (25.4) 10.0 [2.0, 46.0]	23.1 (25.9) 10.0 [2.0, 46.0]	0.12 (0.8)	
Fish	10.4 (14.8) 3.0 [0.0, 17.0]	11.6 (17.4) 3.0 [0.0, 16.0]	0.85 (3.6)	
All groups	12.1 (18.0) 3.0 [0.0, 15.0]	12.9 (19.0) 4.0 [1.0, 14.0]	0.53 (2.5)	*P* = 0.003^+^
Joint space narrowing score				
Borage	11.3 (19.1) 3.0 [0.0, 16.0]	11.7 (18.9) 3.0 [0.0, 18.0]	0.25 (1.6)	*P* = 0.27*
Combination	26.3 (31.0) 16.0 [5.0, 41.0]	27.8 (32.3) 16.5 [5.0, 42.0]	0.96 (2.6)	
Fish	12.7 (20.0) 4.5 [0.0, 15.0]	14.8 (20.9) 5.0 [0.0, 25.0]	1.43 (3.7)	
All groups	15.6 (23.4) 5.0 [0.0, 19.0]	16.9 (24.1) 5.0 [0.0, 25.0]	0.91 (2.9)	*P* = 0.003^+^

*Test of change among groups.

^+^Test of change over time in groups combined.

## Key Messages

Treatment of RA with oils reduced disease activity; disease activity reduction is similar for fish oil, borage seed oil, and the combination of both oils; treatment with oils reduced administration of more toxic therapy.
